# A Scoping Review of Studies Utilizing the Delphi Method in Aging Research from 2018 to 2024

**DOI:** 10.1093/geroni/igaf122.2393

**Published:** 2025-12-31

**Authors:** Aaron Ogletree, Shannon Jarrott, Shelbie Turner, Michelle Demetres

**Affiliations:** Virginia Tech, Blacksburg, Virginia, United States; The Ohio State University, Columbus, Ohio, United States; Weill Cornell Medicine, New York, New York, United States; Weill Cornell Medicine, New York, New York, United States

## Abstract

The Delphi method is a structured process used to obtain expert input on the development of evidence-based tools and resources like quality indicators and clinical guidelines. Despite an estimated 250% increase in the number of studies utilizing the Delphi method since 2014, no published literature has explored its use in aging research. The goals of this scoping review were to characterize applications of the Delphi method in aging research and identify areas for improvement in its application. We searched multiple databases for published literature from January 2018 through November 2024, identified eligible studies using predefined criteria, and extracted key information (e.g., scientific topics and alignment with best practices). We identified 89 Delphi studies in aging research that met inclusion criteria. The most frequent topics included models of care (n = 9), technology (n = 8), and bone health (n = 7). Delphi studies were seldom used to develop materials for subpopulations of older adults (e.g., racial/ethnic minority groups). Experts were mostly clinicians/care providers (n = 78) and researchers (n = 52); older adults (n = 8), unpaid caregivers (n = 3), and advocacy and education groups (n = 5) were least represented. Few studies (n = 9) adhered to guidance on Delphi best practices (e.g., CREDES), highlighting that most studies omitted checklists used to ensure transparent reporting of Delphi conduct. Our findings document trends in use of the Delphi method in aging research and document gaps in representation of experts and focal populations. Opportunities to improve Delphi application in aging research include identification and promotion of best practices for researchers and journals focused on aging.

